# Spinocerebellar Ataxia 7: A Report of Unaffected Siblings Who Married into Different SCA 7 Families

**DOI:** 10.1155/2014/514791

**Published:** 2014-05-04

**Authors:** Fariha Zaheer, Dominic Fee

**Affiliations:** ^1^Department of Neurology, Baylor College of Medicine, Michael E. DeBakey VA Medical Center, Parkinson's Disease Research Education and Clinical Center, Houston, TX 77030, USA; ^2^Department of Neurology, University of Kentucky, Lexington, KY 40536, USA

## Abstract

Two families with spinocerebellar ataxia type 7 are presented. Although there are affected cousins, it is not the sibling parents that transmitted the mutation. It is assumed that the affected families share a common ancestor.

## 1. Introduction

Spinocerebellar ataxia 7 (SCA 7) is a rare disorder [1, 2]. It has a prevalence of less than 1 : 100,00 and accounts for around 2% of all SCAs/autosomal dominant ataxias [2, 3]. As with all genetic conditions, there are regions of increased prevalence. Within the western hemisphere, two regions of increased prevalence of SCA 7, Brazil and Mexico, have been assessed and felt to be due to a founder effect, with a potential/probable European origin of the SCA 7 gene in both [4–6].

We report multigenerational families with SCA 7 in which two unaffected siblings married into separate families, each affected by SCA 7. This did initially confuse assessment and highlights that, at times, two unusual/rare events are present. It is assumed that the two affected families share a remote ancestor.

## 2. Case 1

A 35-year-old female (III:4) was evaluated in our clinic for vision and balance issues. She had history of seizures since the age of 21; these were controlled with Phenobarbital and Phenytoin, which were weaned off after 10 years of therapy. She developed her balance and vision problems at the age of 30. By the age of 33, she had significant unsteadiness, was falling, and could no longer work. She then developed gradually progressive dysarthria and dysphagia. She was evaluated by ophthalmology at the age of 32 and had normal eye exam. By the age of 35, visual acuity was decreased to 20/70 in both eyes with reports of pigmentary mottling of retina. Saccadic eye movements were hypometric in all directions.

Neurologic examination, at that time, revealed appendicular and truncal ataxia. Sensory examination was normal. She had generalized hyperreflexia with downgoing plantars. Over the years, ataxia and vision deterioration gradually progressed. MRI imaging of her brain, at the age of 38 and 45, revealed brainstem and cerebellar atrophy. Genetic testing for DRPLA, SCA 1, and SCA 3 was unremarkable. She had a seizure at the age of 45. Her last evaluation was in her mid-50s. She was blind to all visual stimuli, had slow extraocular movements, and was wheelchair bound. On exam, she had increased tone, hyperreflexia, and upgoing toes; in addition to her upper motor neuron findings, she had cerebellar symptoms such as dysmetria, ataxia, and dysarthria.

**Figure 1:**
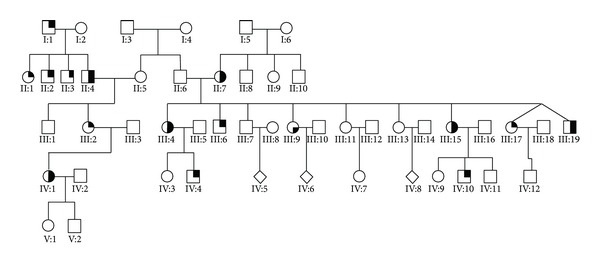
Pedigree of the affected families. Filled upper right quadrant-ataxia; filled lower right quadrant-vision loss/retinal degeneration.

III:4 was one of nine children (Figure 1). She had a daughter, IV:3, in good health and a son, IV:4, who had seizure disorder and balance problems. She had a brother, III:6, who developed difficulties with his balance in his mid-30s. He had no children.

Another brother, III:7, had no symptoms. He had eight children, all healthy. III:4's sister, III:9, had vision problems by her 40s, but no balance issues were reported. She had four children, all of whom were asymptomatic. A sister, III:11, had worn glasses since childhood, but vision had not deteriorated; she did not had any balance problems. She had an asymptomatic daughter. A sister, III:13, was also farsighted but otherwise healthy; she had 2 healthy sons. A sister, III:15, developed vision problems and ataxia in her early 30s. A son, IV:10, had unsteady gait (described later). The last two siblings were twins, III:17 and III:19. III:17 was reported to have mild vision and balance problems beginning in his mid-20s. III:19 died in his early twenties; he was blind by the age of 18 and was wheelchair bound due to falls by the age of 20.

III:4's mother, II:7, developed balance issues in her early 50s. She was reported to have vision problems. II:7 had 3 other siblings (2 brothers and 1 sister) who did not have any history of seizures, vision, or gait difficulties. Their parents were also not reported to have balance or vision problems.

III:4's father did not have any significant health problems. He had a sister, II:5, who was also reported to be healthy. She had a son, III:1, and a daughter, III:2. The daughter, III:2, at age 48, was also evaluated for balance problems. Genetic testing confirmed SCA 7; however, repeat length was not reported. She had a daughter, IV:1, who developed balance and vision problems in her 30s. III:2's father, II:4, 2 paternal uncles, II:2 and II:3, one paternal aunt, II:1, and paternal grandfather, I:1, all had history of balance problems and falls. III:4 and her siblings are first cousins to III:2, but it is not their sibling parents that are affected with ataxia.

## 3. Case 2

IV:10 is the nephew of III:4. He developed unsteady gait and dysarthria at the age of 15.

**Figure 2:**
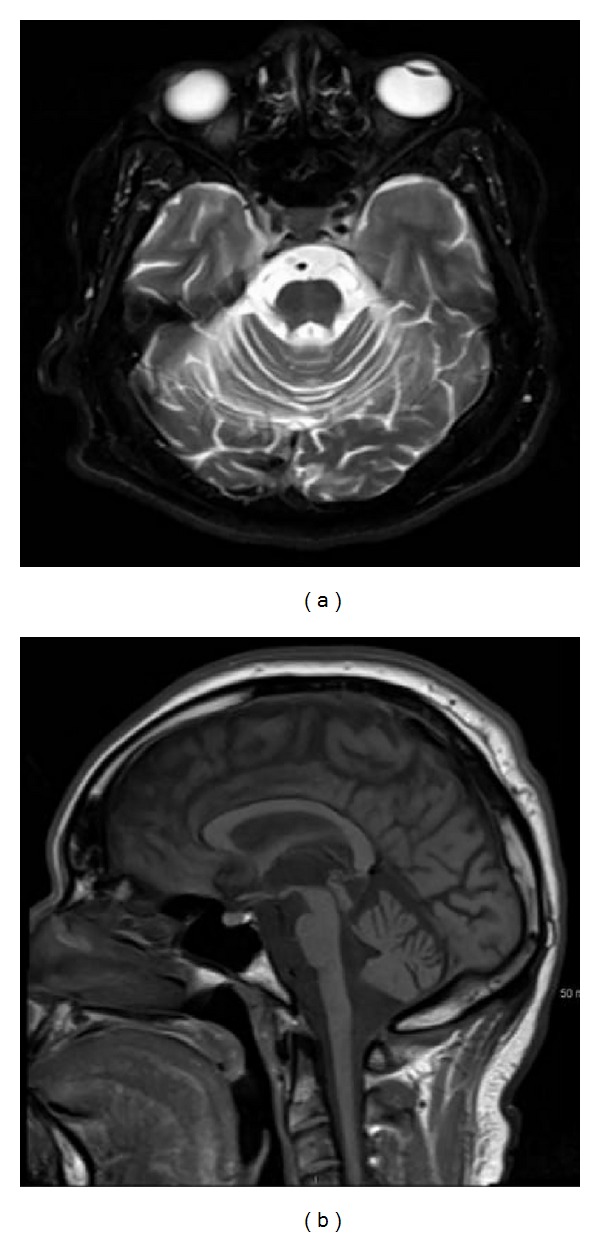
MRI of IV:10, an axial T2 on the left and sagittal FLAIR on the right, demonstrating pontine and cerebellar atrophy.

He had progressive vision deterioration, starting at the age of 18, and was completely blind by the age of 24. His dysarthria gradually worsened over time. MRI head revealed cerebellar and mild pontine atrophy (Figure 2). He was tested for SCA 7 mutation, based on family history and symptom complex. It showed CAG repeat length of 11 in the normal allele and expanded 55 CAG repeat in the other. By the age of 33, he was wheelchair bound due to severe ataxia and blindness. He also had limited eye movements, spastic dysarthria, incontinence, and spasticity in upper and lower extremities with hyperreflexia.

## 4. Comments

Autosomal dominant ataxias are a heterogeneous group of neurodegenerative disorders comprising cerebellar ataxia in combination with other distinct features [7]. Spinocerebellar ataxia 7 is the only autosomal dominant ataxia that presents with unique combination of gait ataxia and progressive vision deterioration [8]. The gene responsible for SCA 7 is located on chromosome 3p12.1, whose mutation is an expanded CAG repeat [9]. The mutated protein, ATXN7, has been shown to cause neuronal loss in cerebellum, regions of brain stem, and retina [8, 10].

Affected individuals in the reported family had cerebellar limb and truncal ataxia, dysarthria, and/or progressive deterioration in vision. These are typical reported symptoms in SCA 7 [11, 12]. In both families, there were individuals who only had either cerebellar gait difficulties or vision deterioration. Ophthalmoparesis was reported in only one patient. III:4 had history of epilepsy in her young adulthood, and her son also had gait difficulties with seizures. Anticipation was definitely noticed in both families.

In most of the reports, maternal transmission is considered to be relatively stable as compared to paternal transmission [13, 14]. More pronounced anticipation has been reported with paternal transmission; in our families, both maternal and paternal anticipation were seen [15, 16]. The first family had predominant maternal transmission with probable increase in size of triple repeats in successive generations. This was reflected by early age of onset in younger generations. On the other hand, the second family had predominantly unstable paternal transmission.

Combination of ataxia and vision loss can be seen in certain other metabolic and genetic disorders like neuronal ceroid lipofuscinosis, Kearns-Sayre syndrome, neuropathy/ataxia/retinitis pigmentosa (NARP), Refsum disease, and so forth [17]. Careful clinical evaluation of patients is essential to avoid costly testing and proceed with appropriate diagnostic and therapeutic options. Genetic counseling along with physical and psychosocial support remains the mainstay of management.
